# Classification of images of wheat, ryegrass and brome grass species at early growth stages using principal component analysis

**DOI:** 10.1186/1746-4811-7-28

**Published:** 2011-09-24

**Authors:** Mahmood R Golzarian, Ross A Frick

**Affiliations:** 1 Department of Agricultural Engineering-Agricultural Machinery, Faculty of Agriculture, Ferdowsi University of Mashhad, Iran; 2 Phenomics and Bioinformatics Research Centre, Australian Centre for Plant Functional Genomics, School of Mathematics and Statistics, University of South Australia, Mawson Lakes, SA, 5095, Australia; 3 School of Mathematics and Statistics, University of South Australia, Mawson Lakes, SA, 5095, Australia

**Keywords:** Image analysis, image segmentation, principal component analysis, weed detection, plant differentiation

## Abstract

Wheat is one of the most important crops in Australia, and the identification of young plants is an important step towards developing an automated system for monitoring crop establishment and also for differentiating crop from weeds. In this paper, a framework to differentiate early narrow-leaf wheat from two common weeds from their digital images is developed. A combination of colour, texture and shape features is used. These features are reduced to three descriptors using Principal Component Analysis. The three components provide an effective and significant means for distinguishing the three grasses. Further analysis enables threshold levels to be set for the discrimination of the plant species. The PCA model was evaluated on an independent data set of plants and the results show accuracy of 88% and 85% in the differentiation of ryegrass and brome grass from wheat, respectively. The outcomes of this study can be integrated into new knowledge in developing computer vision systems used in automated weed management.

## Introduction

Wheat is the most common agricultural crop in southern Australia and annual ryegrass and brome grass are reportedly the two most common weeds in South Australian wheat fields [1]. These weeds are highly competitive, competing with the crop plants for nutrients at an early stage of growth and producing a large seed bank and subsequently a high number of weeds at emergence. They are host to some cereal diseases and can severely affect wheat yield [1]. Management strategies have not been perfected for weedy grasses in contrast to those used for controlling many broadleaf weeds. These plants are similar in appearance to wheat and require several weeks of growth before distinguishing characteristics and vegetative components fully develop [2]. Nevertheless, the early detection of weed invasions and a quick and coordinated response in order to eradicate them are very important before the weeds become too well established and widespread, making control technically and financially unviable. The weeds that are not detected early may require costly ongoing control efforts [3].

The conventional means of manual weed detection is very time consuming, expert-intensive, and costly, even at the early growth stages. On the other hand, early intensive herbicide spraying is not considered an economically and environmentally good option. Therefore, a vision-based and image analysis method was proposed as a cost-effective and site-specific replacement method for weed detection. Digital image analysis has found recent applications in plant biology, plant taxonomy and precision agriculture [4-16]. Perez et al (2000) used a colour RGB camera to detect broadleaf weeds between rows of a cereal crop. Shape analysis was applied for the plants particularly between the rows to detect the weeds. Morphological techniques have been successful to separate broadleaf regions from narrow-leaf plants [17]. A simple approach is to apply a successive erosion process by which the narrow-leaf plants are removed leaving only broadleaf plants. However, other morphological features have been used to separate broadleaf crop plants from narrow-leaf weeds, or vice versa [18].

Hemming and Rath [19] developed a fuzzy-logic computer vision method to differentiate broadleaf cabbage and carrots from narrow-leaf weeds. They used some colour features and some shape features such as area, length/width and convexity for classification. Tillet et al. [20] developed a vision-based, small autonomous vehicle to detect transplanted cauliflowers and spray the weeds. They used size and some shape features to pick the crop plants aligned in a row fashion. These studies mainly focus on broadleaf mature plants of more than three to five leaves.

In spite of these efforts, there has been little in the way of theoretical advances in developing a robust general method for combining colour, morphological and textural features for crop-weed classification. In particular, most of the shape-based classification approaches have been developed for broadleaf plant classification. This present study focuses on the crop plant (wheat) and two prominent weeds encountered in Australian farming all of which are narrow-leaf plants. Our aim is to fill in the gaps in our knowledge of the use of combinations of visual properties for the differentiation of narrow-leaf plant species.

Having said that, vision-based recognition of grass species is still considered a difficult task. The difficulties are less challenging when distinguishing narrow-leaf plants from broadleaf weeds or using spectral characteristics of certain crop and weed species [8,19,21-25]. Little effort has been made in the area of identification of narrow-leaf grass plants based on the visual properties to guide their identification from the images using digital image processing techniques. The main objective of this study is to develop a vision-based method for identifying wheat from common weed species from their images.

## Materials and methods

A vision-based approach to describe a plant involves defining and measuring some specific visual characteristics such as colour (e.g. red, green, and blue), shape (e.g. area, perimeter, major and minor axis) and texture features (e.g. intensity contrast). In this experiment, quantitative analogues of these features are extracted from the images of plant species using image processing techniques, and Principal Component Analysis is employed to extract a descriptor for differentiating between plant species.

### Acquiring and processing the images

**Figure 1:**
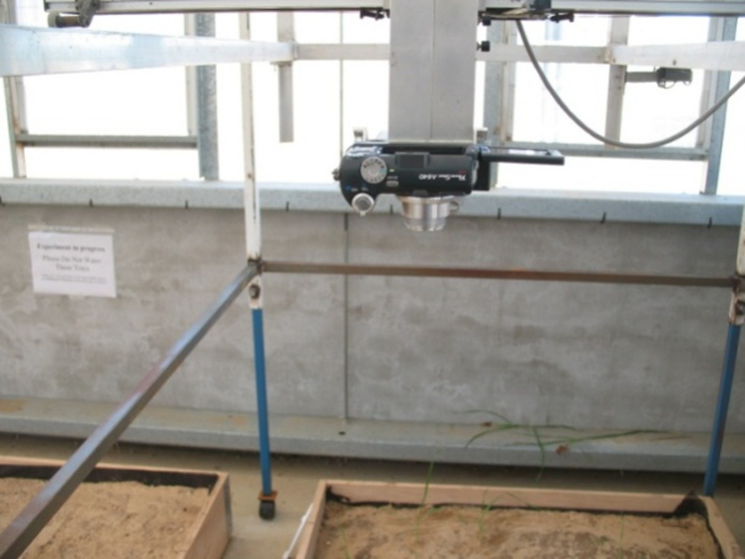
**Experimental planting box in greenhouse and imaging set-up, frame and camera system used to acquire images**.

The images used for this study were of three plant species cultivated in a 1500 mm × 1000 mm box in a greenhouse facility from the School of Natural and Built Environments at the University of South Australia, (Figure 1). Within each planting box, 36 plant positions were arranged spaced 150 mm apart and at 125 mm to the edge of box. Thirty six seeds (12 per species) were planted in each box. The seeds were obtained from the seed bank of the Department of Plant Science at the University of Adelaide. Temperature was controlled for 18°C during the days and 16°C for nights, and humidity was in the range of 50-60%. The experiment was conducted from December 2007 until the end of February 2008 (summer 2008 in Australia).

Plant seedlings were imaged regularly every three days following first emergence. The images were taken between 11 AM and 1 PM when the light intensity was high and in the range of [8000-12000] lux. A Canon PowerShot A640 (Canon, Inc) with 1/1.8" sensor size and focal length of 7.3 mm was used for imaging. The images were of the size of 3648 × 2736 pixels and taken from the top view of the plant seedlings at a distance of 1000 mm. The field of view was 980 mm × 720 mm and the pixel ground resolution was 0.266 mm/pixel (0.07 mm^2^/pixel).

We developed an algorithm in Matlab^® ^(Mathworks, Natick, MA, USA) to display the image and zoom on to each part of the image containing a plant systemically and the image of each individual plant was cropped from the full image manually for further processing. Not all seeds germinated. The successful seeds provided us with the total 286 images of individual plants during their 1-4 leaves growth period. From this number, we obtained 118 images of wheat, 122 of Brome grass and 46 of ryegrass species. Of the 286 images, 57 images (20%) were randomly selected and put aside for testing a method developed using the remaining 229 images (80%).

**Figure 2:**
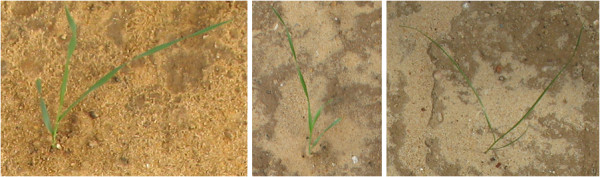
**(Left to right) Seedlings of wheat, brome grass and ryegrass at their three leaf growth stage**.

Examples of images of individual wheat, ryegrass and brome grass seedlings are shown in Figure 2.

**Figure 3:**
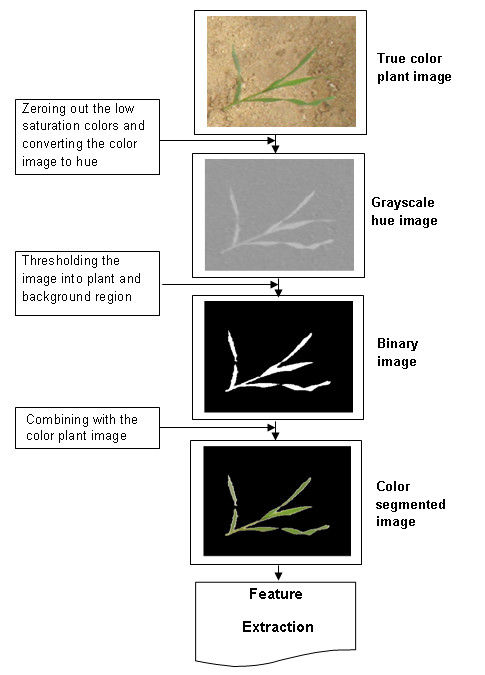
**Process flow of image processing steps used in feature extraction from the plant images**.

In order to extract visual characteristics (or features) of plants from the images, the plant regions needed to be separated from the background by a segmentation process. This was accomplished by converting each true colour image to a grayscale hue image first. Grayscale hue images provide high contrast between plant regions and non-plant background, making the segmentation process easier and more accurate. A hue image is the same size as the actual image with each pixel containing a value in the range of 0° to 360°, representing the position of the colour on the hue circle. Pixels with low colour saturation were zeroed out before the segmentation process [26]. From previous work it was known that the pixels of green plant regions have hue values in the range of 54° to 154° with the minimum noise error [27,28]. These values were used as the thresholds to binarise the hue image. The resulting black and white image was used as a mask and combined with the true colour image to yield a colour segmented image ready for further processing. The flow chart in Figure 3 shows the image processing steps used before extraction of the plant's visual features. We developed all the routines and codes required for the image processing steps including contrast enhancement, image segmentation and feature extraction with Matlab's image processing toolbox.

The plant image shown in Figure 3 is typical of the images used in this application. In spite of the rather coarse appearance the resulting segmented images were adequate for subsequent analysis.

In theory, there are a large number of visual characteristics of plants which can be extracted from their images. However, in this study, an expert-based approach was followed to select the optimum relevant features [29-31]. The expert uses a combination of colour, texture, and shape features, to distinguish between plants, but even the expert has difficulty with plants in the two to four leaf stage of growth. Some of these features (such as a blue tinge in the leaf, or the length-to-width ratio of the leaf) are fairly easily described, and may be quantifiable. Others (such as "texture" in some generalized sense) are not so easily described. In the field, in some cases, in addition to the visual properties of the plants, close inspection of the shape of ligules and auricles, and the colour of the plant base, are required to discriminate weed species from wheat. It was hypothesized, however, that the image of a single leaf may contain more information than a human eye can easily detect and therefore digital information may provide a greater potential for differentiation between these plants. Out of the many combinations of colour intensity values and geometrical parameters which could have been used, we restricted our attention to those which mimicked the response of the expert human eye, in the expectation that these would most likely yield distinguishing characteristics in the images. Table 1 summarizes the features the weed experts suggested as useful features in differentiating the selected plant species and some equivalent features from an image processing perspective. The full definition and expressions of the equivalent image processing features are selected from among many possible features given in Table 2.

**Table 1:** Selected features illustrating an expert-based approach to plant identification

Species	Visual features used by experts	Relevant image processing features	Feature type
Wheat	Green leaves	g_i_, EGI,	Colour
	Width and length ratio	Width, Waddle Disk Ratio	Shape
	Hairless leaves	Uniformity, Entropy	Texture

Brome grass	Reddish at the base,	ERI, r_i_, EBI	Colour
	bluish green leaves	b_i_, g_i_, EGI, b_i_, EBI	Colour
	Width and length ratio	Width, Waddle Disk Ratio	Shape
	Small hair on the leaves,	Uniformity, Entropy	Texture
	shiny leaves	Uniformity, Entropy	Texture

Ryegrass	Reddish base and	b_i_, EBI, RBI	Colour
	different green colour	g_i_, EGI	Colour
	Narrower leaves	Width, Waddle Disk Ratio	Shape
	Hairless shiny leaves	Uniformity, Entropy	Texture

**Table 2:** Features used for differentiation

Feature	Definition
r_i_	R/(0.2989 * R + 0.5870 * G + 0.1140 * B)
g_i_	G/(0.2989 * R + 0.5870 * G + 0.1140 * B)
b_i_	B/(0.2989 * R + 0.5870 * G + 0.1140 * B)
RBI	(r_i_-b_i_)/(r_i_+b_i_)
ERI	(r_i_-g_i_)×(r_i_-b_i_)
EGI	(g_i_-r_i_)×(g_i_-b_i_)
EBI	(b_i_-g_i_)×(b_i_-r_i_)
W	2 × erosion steps
WDR	W/Waddle Disk Diameter
Uniformity (U_t_)	$\overset{L-1}{\underset{i=0}{\sum }}p^{2}\left(z_{i}\right)$
Entropy (E_t_)	$-\overset{L-1}{\underset{i=0}{\sum }}p\left(z_{i}\right)\;log\;p\left(z_{i}\right)$

The three colour components of R, G and B are the intensity values in the range of 0-255 of the colour channels of Red, Green and Blue, respectively. The factors r_i_, g_i _and b_i _are colour factors normalized by the grayscale intensity of the pixel. Grayscale intensity is the attribute of light that expresses the amount of illumination and is computed as a weighted sum of the R, G, and B components [32]. Normalization of colours, achieved by dividing the pixel value of a colour by the pixel's grayscale intensity, reduces the effect of illumination on the pixel colour values.

The ratio of the width (W) over Waddle disk diameter was used for the first time and defined as "Waddle disk ratio" (WDR). To measure the width (W), the plant region in the binary image was eroded from the border pixels inwards iteratively and until the whole region disappeared. The kernel in the morphological operator was selected as a 3 × 3 square which allows the removal of one pixel-wide layer around the plant region per iteration. The width is calculated as twice the number of iterations required to achieve this.

The Waddle disk diameter is the diameter of a circle with the same area as the plant region in the binary segmented image. The Waddle disk ratio, then, is a dimensionless parameter which by definition measures the roundness (as opposed to linearity) of the leaf area.

**Figure 4:**
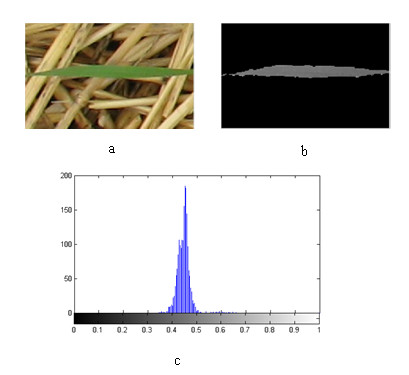
**Texture analysis: a) original plant leaf image; b) gray image of the segmented plant leaf c) histogram of the gray-shade intensity of the whole plant leaf region shown in b**.

In addition to the colour and shape features, texture features of plant regions have also been extracted from their grayscale images [33-36]. A grayscale image is a monochrome image whose pixel values are grayscale intensity. Texture, interpreted in quantitative terms, is variability in reflectance of the surface of the region of interest. In an image, texture appears as variation in grayscale values. In this study, two common statistical histogram-based texture features of "Uniformity" and "Entropy" were extracted from the histogram of the region of interest (Figure 4).

Uniformity is a maximum when all the gray levels are equal and minimum when the histogram has equal proportions. Entropy measures the degree of randomness and it is a maximum when the histogram has equal proportions. In Table 2, the symbol *p(z*_*i*_*) *is the proportion of pixels having a given intensity level *z*_*i*_, and L is the number of possible intensity levels [37].

### Principal Component Analysis

Once the visual features were extracted, Principal Component Analysis was employed to extract a pattern for differentiating between plant species. Principal Component Analysis (PCA) is an algebraic technique (eigen-decomposition) in which combinations of correlated variables are selected as explaining the variability in observations between images. The resulting principal components have greatly reduced (ideally, zero) correlation. By this means a smaller set of relatively uncorrelated variables may take the place of a larger set of correlated variables [38]. The coefficients in the combination that give rise to the components are known as loadings, while the eigenvalues measure the variability between images associated with each component.

The manner in which PCA creates combinations of measured variables has an intuitive appeal, in that the process in a certain way mimics the cognitive process used by the expert to distinguish plants. The parts to the complete computational process are, firstly, creating quantitative analogues to the visual features; secondly, selecting those which display measurable differences between plants; and finally, finding the appropriate combination of these features to distinguish one plant from another.

In this study, we used SPSS software package (version 17, IBM, Chicago, Illinois, USA) to conduct PCA and the results were verified using Matlab.

### Variable selection for PCA

The correlation matrix exhibited a number of high positive and negative correlations, which are an indication of redundant information (Table 3). As can be seen from the correlation table, several feature pairs are highly correlated. For example, Uniformity and Entropy are highly negatively correlated, therefore only one of these features is selected. All the variables with an absolute correlation value of < 0.7 and only one of the highly correlated variables (|correlation value| ≥ 0.7) were selected for further consideration. As a result six relatively uncorrelated features were selected for building the PCA model, namely, r_i_, RBI, EBI, width, Waddle Disk Ratio, and uniformity.

**Table 3:** Correlation matrix (shaded cells show the high correlation)

	r_i_	g_i_	b_i_	RBI	ERI	EGI	EBI	W	WDR	U_t_	E_t_
r_i_	1.0										
g_i_	**-0.7**	1.0									
b_i_	-0.3	-0.5	1.0								
RBI	0.4	**-0.9**	0.6	1.0							
ERI	**-0.7**	**1.0**	-0.4	**-0.9**	1.0						
EGI	0.4	0.4	**-1.0**	-0.5	0.3	1.0					
EBI	0.5	0.3	**-1.0**	-0.5	0.2	**1.0**	1.0				
W	-0.6	0.6	0.0	-0.4	0.6	-0.1	-0.2	1.0			
WDR	0.4	-0.3	-0.1	0.3	-0.3	0.2	0.2	-0.3	1.0		
U_t_	0.3	-0.2	-0.1	0.2	-0.2	0.1	0.1	-0.2	0.5	1.0	
E_t_	-0.4	0.2	0.2	-0.2	0.3	-0.2	-0.2	0.3	-0.5	**-0.9**	1.0

### Building the PCA model

The PCA with Varimax rotation was conducted on the correlation matrix to assess the underlying structure of the six features for the plant species differentiation. Varimax is an orthogonal rotation method which is employed to rotate components while keeping them orthogonal and uncorrelated. Varimax attempts to maximize the dispersion of loadings within components. Thus, this method loads highly a smaller number of variables onto each factor resulting in more interpretable clusters of components [39].

**Figure 5:**
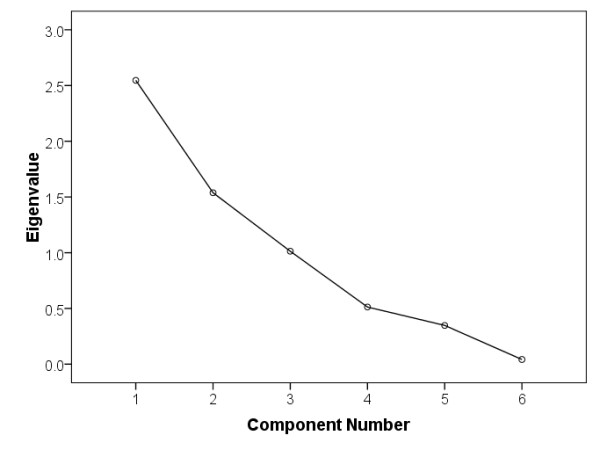
**Scree plot of the PCA model**.

Principal components from the model building set of plant images (= 229 samples) were computed from the eigenvectors. Figure 5 shows the scree plot of the six components. A scree plot is a plot of the eigenvalues, in descending order of magnitude, and helps the analyst visualize the relative importance of the components [40]. Three Varimax rotated principal components were selected using the combination of scree plot and "Kaiser criterion". The Kaiser criterion is the most widely used answer to the question on how many factors to retain. This criterion says only those components with eigenvalues greater than 1 should be retained. However, this criterion sometimes retains too many and sometimes too few factors and in practice a scree plot is also used to decide if the best number of factors has been chosen. In the scree plot, the first few factors before the tail begins are often chosen as the best factors [41]. Having examined the scree plot (Figure 5) and considering the eigenvlaue of the third factor falling outside Kaiser's criterion by a tiny margin, we included the third factor in the PCA model.

The first principal component accounts for 42% of the total standardized variance in the data set. The second component accounts for 26% and the third principal component accounted for 17% of variability between the images (Table 4). As can be seen, 84% of the variability between plant images is explained by these three components.

**Table 4:** Total variance explained by the principal components obtained from the model building set (n = 229)

Component	Initial Eigenvalues		
	
	Total	% of Variance	Cumulative %
1	2.50	41.74	41.74
2	1.51	25.21	66.95
3	1.00	16.66	83.61

These three principal components were created as linear combinations of the original features. The loadings used to create the linear combinations are given in the coefficient matrix (Table 5).

**Table 5:** Component score coefficient matrix for the model (n = 229)

	Component		
	
	1	2	3
r_i_	**0.482**	0.127	-0.081
RBI	0.251	**-0.515**	-0.022
EBI	0.183	**0.602**	-0.018
W	**-0.470**	0.055	0.114
WDR	-0.088	-0.020	**0.574**
U_t_	-0.180	0.011	**0.651**

The first component, which seemed to identify the colour feature of redness along with the shape feature of width, loads most strongly and positively on the redness and negatively on the width. The second component which seemed to identify the contrast between red and blue is comprised of two colour features with high (that is, numerically large) loadings in the second column. The two features RBI and EBI have almost the same loadings in this component but of opposite sign. The third component, which seemed to represent a combination of texture and shape, was composed of the two features with high loadings in the third column of the table. The Waddle Disk Ratio had its highest loading on the third component. The linking of two unrelated features in one single component was unexpected. It seems that there is an intrinsic relationship between these features which differs from plant to plant in a way not obvious to the human eye.

The main source of variability between the plant images is the result of some plants having high values of the principal components and some not. Having built the model, we expect that the components will provide the independent explanations of the differences between the plant images.

## Results and discussion

### Model performance in plant differentiation

To calculate the component score for each plant image, the factor loadings were multiplied by the values of the visual features obtained from each image of the plant. The calculated component scores were then used as response variables in the procedure Analysis of Variance (ANOVA) with the plant type as the categorical level, and statistically significant differences were found between the three principal components [38,42]. The ANOVA table (Table 6) shows that the variations between images of different plant species are much greater than the variations between the images of the same plant for principal components 1 to 3 (PC1, PC2 and PC3). Therefore, we expect that these PCs are able to distinguish between the images of the three plant species.

**Table 6:** ANOVA table comparing plant type on scores of PC1, PC2 and PC3

		Sum of Squares	df	Mean Square	F	**Sig**.
PC1	Between Groups	47.208	2	23.604	29.506	.000
	Within Groups	180.792	226	.800		
	Total	228.000	228			

PC2	Between Groups	22.985	2	11.493	12.669	.000
	Within Groups	205.015	226	.907		
	Total	228.000	228			

PC3	Between Groups	30.213	2	15.106	17.261	.000
	Within Groups	197.787	226	.875		
	Total	228.000	228			

The statistical significance of differences between the images of plant species within each principal component was tested with Bonferroni post hoc tests [38,43]. Post hoc Bonferroni analysis (Table 7) indicated that wheat and ryegrass differed significantly in their values of PC 1 as did brome grass and ryegrass. However, there was no significant difference between the mean of PC1 for wheat and that for brome grass. Therefore, the first component can be used to distinguish ryegrass from the other two grasses. Likewise, there was also significant mean difference on the values of PC2 between brome grass and the rest. Therefore, this component can be used to distinguish brome grass from the other two grasses. The test also showed the mean difference between the values of PC3 for ryegrass and the other two plants was significant (P < 0.005).

**Table 7:** Bonferroni post hoc tests

Dependent Variable	(I) plant type	(J) plant type	Mean Difference (I-J)	Std. Error	**Sig**.
PC1	Wheat	Brome grass	-.02	.13	1.00
		Ryegrass	-1.23*	.17	.00
	
	Brome grass	Wheat	.02	.13	1.00
		Ryegrass	-1.21*	.17	.00
	
	Ryegrass	Wheat	1.23*	.17	.00
		Brome grass	1.21*	.17	.00

PC2	Wheat	Brome grass	-.60*	.14	.00
		Ryegrass	.10	.19	1.00
	
	Brome grass	Wheat	.60*	.14	.00
		Ryegrass	.70*	.18	.00
	
	Ryegrass	Wheat	-.10	.19	1.00
		Brome grass	-.70*	.18	.00

PC3	Wheat	Brome grass	.16	.14	.74
		Ryegrass	1.04*	.18	.00
	
	Brome grass	Wheat	-.16	.14	.74
		Ryegrass	.89*	.18	.00
	
	Ryegrass	Wheat	-1.04*	.18	.00
		Brome grass	-.89*	.18	.00

**Figure 6:**
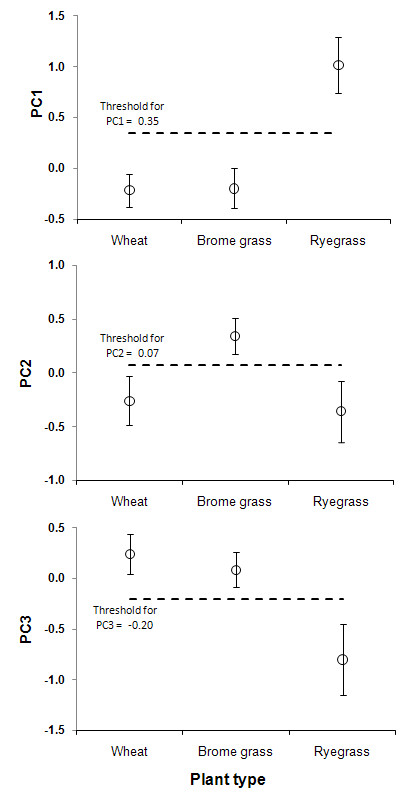
**Component scores extracted from PCA versus plant types (error bars indicate 95% confidence intervals)**.

We were now able to set up a method that can be used to distinguish crop wheat plants from ryegrass and brome grass weed plants. This method used scores for three components as the classifiers and a threshold value for each classifier. The threshold values were calculated from the confidence intervals mentioned in the detailed table of descriptive statistics (Table 8). For instance, for PC1, a threshold between the upper bound for wheat and lower bound for ryegrass is the threshold value used to differentiate these two plant species. For PC2, a value in between the lower bound of the interval for wheat and upper bound of that for brome grass is the threshold value for separating wheat and brome grass. The threshold values were used later in the validation process as the selection criteria for testing if the component scores obtained from a new dataset could in fact differentiate plant species accurately. The threshold values for all three components are shown on the diagrams in Figure 6.

**Table 8:** Descriptive statistics for principal components

Component	Plant type	N	Mean	Std. Deviation	Std. Error	95% Confidence Interval for Mean	
						
						Lower Bound	Upper Bound
PC1	Wheat	87	-.21	.76	.08	-.37	-.05
	Brome grass	104	-.19	1.01	.10	-.39	.00
	Ryegrass	38	1.02	.82	.13	.75	1.29
	Total	229	.00	1.00	.07	-.13	.13

PC2	Wheat	87	-.26	1.07	.11	-.49	-.03
	Brome grass	104	.35	.88	.09	.17	.52
	Ryegrass	38	-.35	.86	.14	-.64	-.07
	Total	229	.00	1.00	.07	-.13	.13

PC3	Wheat	87	.24	.92	.10	.05	.44
	Brome grass	104	.09	.90	.09	-.09	.26
	Ryegrass	38	-.80	1.06	.17	-1.15	-.45
	Total	229	.00	1.00	.07	-.13	.13

### Validating the method

Having established a system of principal components that discriminates the three plant species from their images it becomes necessary to validate the system on an independent data set. The colour, shape and texture features of the images in the testing dataset (= 57 samples) were converted into the principal component scores using the variable loadings presented in Table 5. Then the computed component scores were compared with the threshold values given for each component shown in Figure 6. The accuracy of components in differentiating plant species was calculated by dividing the number of correctly discriminated plants by the total number of plants (Table 9).

**Table 9:** Discrimination accuracy for the three components

Component	Used in discrimination of	Accuracy (%)
PC1	wheat and ryegrass	82.4
PC2	wheat and brome grass	84.6
PC3	wheat and ryegrass	88.2

The first component, which has succeeded in discriminating ryegrass from wheat, has high loadings on normalized red r_i _and width W (negative), with some contribution from the red-blue contrast feature RBI. PC1 yielded higher values for ryegrass than for wheat. Evidently the process has been able to detect that ryegrass plants have more red and less blue in the colour, and narrower leaves, than wheat plants, in the early growth stages.

The second component, which has distinguished brome grass from wheat, has high loadings on the red-blue contrast feature RBI (negative) and the excess-green feature EGI. PC2 yielded higher values for brome grass than for wheat. The process has been able to detect some subtle differences in the colours of these plants, not easily discerned by the human eye, to do with the green and blue content of the leaf colour.

The third component, which has been even more successful in discriminating ryegrass from wheat, has high loadings on the Waddle disk ratio and the uniformity. PC3 yielded larger negative values for ryegrass than for wheat. This indicates that the process has detected that ryegrass plants have a less uniform leaf surface, combined with more linearity in the leaf shape, than wheat plants, at this stage of growth.

The differentiation obtained by these three components approaches that achievable by a trained observer. With higher image resolution enabling better quantitative measures of texture and colour the accuracy is likely to improve significantly, leaving only biological variation as the source of error. The image processing and PCA of themselves are essentially error neutral.

## Conclusions

Early detection of weeds followed by quick and appropriate actions to remove the weeds is an important part of weed management because if the weeds become too well established and widespread their control becomes technically and financially impossible. However, identification of and dealing with narrow leaf weeds in wheat farms can be a frustrating experience particularly during early growth stages, and it would be desirable to have a machine-based method for identifying and dealing with them.

The first step in developing such a method is to automate the identification of individual plants. This study demonstrates that it is possible to differentiate greenhouse-grown wheat from ryegrass and brome grass based on their images with identification accuracy of 88% and 85%, respectively. Given the difficulties of identification of these very similar narrow-leaf species up to the four leaf stage, the achieved accuracies in discriminating brome grass and ryegrass from wheat indicate that automatic identification is feasible. These results were obtained using the images of the plant species grown in the greenhouse environment. As future work, we would like to test our method on images taken under field conditions. The ultimate goal, however, will be to develop the machine vision technology so that crop and weeds may be identified in situ, using a process similar to that outlined here, perhaps using pre-determined thresholds for the discriminating components. A simple application would be in the early identification of weed infestation in a recently planted crop. But it is surely not too futuristic to envisage a machine, equipped with a high definition camera, a fast computer, and other appliances, progressing through a crop while identifying and dealing with individual weeds.

## Competing interests

The authors declare that they have no competing interests.

## Authors' contributions

MRG conceived the study, conducted the glasshouse experiment, acquisition and processing of the image data, carried out the preliminary data analysis, and wrote the first draft of manuscript. MRG and RAF cooperated in developing the PCA model, the further data analysis and interpreting the results. RAF assisted in reading, editing, writing and approving the final manuscript. All authors read and approved the final manuscript.
